# X-Ray-Induced Modification of the Photophysical Properties of MAPbBr_3_ Single Crystals

**DOI:** 10.1021/acsami.1c16072

**Published:** 2021-12-01

**Authors:** Giovanni Armaroli, Laura Ferlauto, Ferdinand Lédée, Matilde Lini, Andrea Ciavatti, Alessandro Kovtun, Francesco Borgatti, Gabriele Calabrese, Silvia Milita, Beatrice Fraboni, Daniela Cavalcoli

**Affiliations:** † Department of Physics and Astronomy, University of Bologna, Viale Berti Pichat 6/2, 40127 Bologna, Italy; ‡ Interdepartmental Center for Industrial Research of the University of Bologna (CIRI-MAM), Viale Risorgimento 2, 40136 Bologna, Italy; § Institute of Organic Synthesis and Photoreactivity—(CNR-ISOF), Via Gobetti 101, 40129 Bologna, Italy; ∥ Institute for Nanostructured Material Study (CNR-ISMN), Via Piero Gobetti 101, 40129 Bologna, Italy; ⊥ Institute for Microelectronics and Microsystems (CNR-IMM), Via Piero Gobetti 101, 40129 Bologna, Italy

**Keywords:** hybrid lead halide perovskites, methylammonium lead bromide, ionizing radiation, surface photovoltage spectroscopy, X-ray photoelectron spectroscopy, excitons

## Abstract

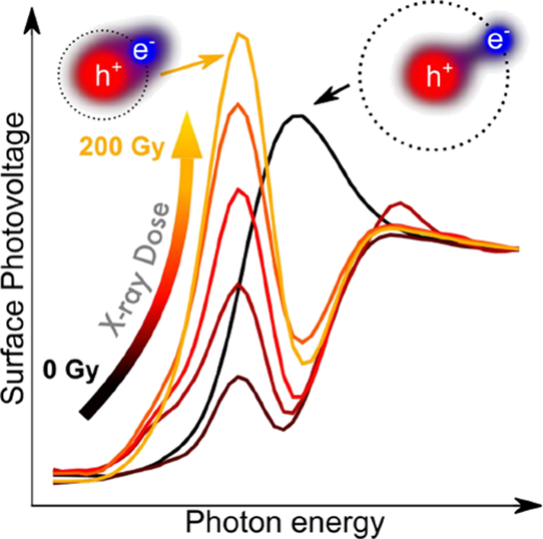

Methylammonium lead tribromide (MAPbBr_3_) perovskite single crystals demonstrate to be excellent direct X-ray and gamma-ray detectors with outstanding sensitivity and low limit of detection. Despite this, thorough studies on the photophysical effects of exposure to high doses of ionizing radiation on this material are still lacking. In this work, we present our findings regarding the effects of controlled X-ray irradiation on the optoelectronic properties of MAPbBr_3_ single crystals. Irradiation is carried out in air with an imaging X-ray tube, simulating real-life application in a medical facility. By means of surface photovoltage spectroscopy, we find that X-ray exposure quenches free excitons in the material and introduces new bound excitonic species. Despite this drastic effect, the crystals recover after 1 week of storage in dark and low humidity conditions. By means of X-ray photoelectron spectroscopy, we find that the origin of the new bound excitonic species is the formation of bromine vacancies, leading to local changes in the dielectric response of the material. The recovery effect is attributed to vacancy filling by atmospheric oxygen and water.

## 1 Introduction

Since the publication of two seminal papers in 2012,^1,2^ the research on organometal halide perovskites (OHPs) for optoelectronic applications has been growing at a rapid pace. These materials show remarkable properties, such as tunable bandgap, long charge carrier diffusion length, defect tolerance, high absorption coefficient, and low-cost fabrication.^3,4^ These are the main reasons of interest for their exploitation in research domains ranging from solar cells and photovoltaic to radiation detection for security and medical applications.^5^ Their chemical composition is generally identified as ABX_3_ where A is a monovalent organic cation (e.g., methylammonium, CH_3_NH_3_^+^, or formamidinium, HC(NH_2_)_2_^+^), B is a divalent metal cation (e.g., Pb^2+^ or Sn^2+^), and X is a halogen anion (e.g., I^–^, Br^–^, and Cl^–^).

Among several possible applications for OHPs, the detection of ionizing radiation (e.g., X-ray and gamma ray) has recently attracted the attention of the research community. The applications of X-ray detectors range from medical imaging and safety screening to quality control in material industry and shipping inspections. Gamma-ray detectors are instead exploited in fields such as radiological security and nuclear defense.^6−8^ In particular, methylammonium lead tribromide perovskite (MAPbBr_3_) single crystals demonstrated very promising results for high-sensitivity X-ray and gamma-ray detectors, combining high stopping power, due to the presence of Pb, with low trap density, high charge collection efficiency, small dark current density, and high bulk resistivity.^9,10^ MAPbBr_3_ single crystals, with their easy and low-cost fabrication process, could indeed be a valid alternative to the current state-of-the art materials for room-temperature solid-state direct detection, such as silicon or cadmium zinc telluride, which are still affected by severe limitations such as high energy consumption and expensive growth facilities for their processing.

Despite the vivid interest of the research community on these materials, MAPbBr_3_ and all other OHPs still suffer from major issues such as limited long-term stability and degradation, which currently impede their usage in commercial devices.^11^ Defects and ion migration play a relevant role in material’s degradation and stability,^12^ but an in-depth understanding of the relationship between OHP electronic and photophysical properties and the presence of defects and charge recombination is somehow still lacking. Ion migration also affects dielectric response, excitonic and polaronic states. Exceptionally high values of the dielectric constant, indicating strong screening of charged carriers, have been considered as the reason for preventing photocarrier trapping and recombination.^13,14^ Exciton binding energy in these materials has been proven very difficult to experimentally determine even for largely studied materials like MAPbI_3_ and MAPbBr_3,_ due to the possible effect of polaron formation and dynamic lattice disorder.^13^ As a consequence, a widespread range of exciton binding energies has been reported for MAPbBr_3_ in the literature,^15,16^ ranging from 14 to 90 meV.

Notwithstanding the above mentioned interest for OHPs as X-ray detectors,^6^ the effect of strong and prolonged irradiation by X-rays on these materials is still not clarified, as only a few studies on ionizing radiation effects have been published. Xu et al.^17^ have investigated gamma radiation effects in MAPbBr_3_, finding relevant structural modifications after high dose irradiation (in the kGy range). Several studies reported surface degradation with formation of metallic lead on MAPbBr_3_ after X-ray^18^ or electron^19^ irradiation. Despite these results, other groups reported OHP solar cell operation to be stable even after high dose irradiation, demonstrating an excellent radiation hardness of these devices.^20,21^

The present manuscript aims to assess the effects of X-ray irradiation in the range of interest for diagnostic applications^22^ (up to hundreds of Gy) on the optoelectronic properties of MAPbBr_3_ single crystals. We carried out irradiation in air with a medical imaging X-ray tube to simulate real-life conditions in a medical facility. With this aim, we grew MAPbBr_3_ single crystals using a seed-assisted inverse temperature crystallization method^23,24^ and characterized the effects of exposure to X-ray radiation by means of surface photovoltage spectroscopy, photoluminescence spectroscopy, X-ray diffraction, and X-ray photoelectron spectroscopy.

## 2 Experimental Methods

### 2.1 Growth of MAPbBr_3_ Single Crystals

The crystal growth followed the seed-assisted inverse temperature crystallization method.^23,24^ Precursor solution was prepared by dissolving methylammonium bromide (CH_3_NH_3_Br) and lead(II) bromide (PbBr_2_) in *N*,*N*-dimethylformamide (DMF) at a concentration of 1 mol/L in a 1:1 molar ratio. All precursors and solvents were purchased from Sigma Aldrich. The solution was stirred for 4 h and filtered through 0.22 um PTFE filters. Precursor solution was heated up to 85 °C in an oil bath at a rate of 3 °C/min, allowing the formation of small crystal seeds that were immediately taken out of the solution, dried, and stored. In the second step of crystallization, a new precursor solution was heated up to 55 °C in an oil bath at a rate of 3 °C/min. Once the temperature was reached, a seed was immerged in the solution and the vial was heated up to 85 °C at a rate of 5 °C/h, allowing the growth of the seeds into crystals of several millimeters in size (typical dimensions 5 mm × 5 mm × 2 mm). A postgrowth process consisting in washing the crystals in six solutions of perovskite solvent (DMF) and antisolvent (chlorobenzene) with decreasing DMF concentration allowed to chemically clean the surface from excess of nano/microcrystallites.

### 2.2 Surface Photovoltage Spectroscopy (SPS)

The measurements were performed in the metal–insulator–semiconductor configuration, where a capacitor structure is formed by positioning an indium-tin-oxide (ITO) electrode in front of the sample surface and by using air as the dielectric. The sample was grounded through a back contact of silver paste. The surface photovoltage (SPV) signal was then capacitively acquired by the ITO electrode connected in series to a 1GΩ resistor, preamplified through a Femto DLPVA voltage amplifier, and finally measured with a Stanford SR 830 lock-in amplifier. The white light from an OSRAM 150 W Xe lamp was monochromated with an SPEX 500 M spectrometer chopped at 20 Hz with a 300CD model optical chopper by Scitec Instruments.^25^ SPV signal was normalized to the lamp intensity by dividing the measured SPV signal by the incident photon flux for each photon energy. The intensity of each curve was also normalized to its value at continuum (around 2.4 eV) in order to align the continuum absorption and compare the relative change in the intensity of the excitonic absorption.

### 2.3 Photoluminescence (PL) Spectroscopy

Measurements were performed at room temperature and in ambient conditions by illuminating the sample with a 375 nm PicoQuant diode laser run at 100 μW power and pulsed at 100 MHz. The PL signal was filtered with a 400 nm long-pass filter in order to eliminate laser reflections and was acquired at a 45° angle with respect to the impinging laser direction using a Thorlabs CCS200/M spectrometer.

### 2.4 X-Ray Irradiation

X-ray irradiation was performed with a Hamamatsu L12161-07. A tungsten target X-ray tube was run at an accelerating voltage of 150 kV and a tube current of 500 μA. The sample was placed at 9 cm distance from the tube, resulting in a dose rate of 72 mGy/s, previously calibrated with a commercial X-ray detector. Based on this value, the exposure time was calculated in order to irradiate the sample with the desired dose.

### 2.5 X-ray Photoelectron Spectroscopy (XPS)

MAPbBr_3_ crystals were fixed on conductive carbon tape immediately after growth. The measured area was selected with an XPS analyzer (Phoibos 100, Specs, Germany) on a 3 mm diameter spot. The X-ray source was an Mg Kα emission (1253.6 eV) with a constant power of 125 W. The X-ray spot size was ca. 20 mm diameter, while the photocurrent measured from the sample was ca. 200 nA. Photoelectrons were collected along the surface normal. The base pressure during the measurement was 1.5 × 10^–8^ mbar. All spectra were calibrated to the position of C 1s corresponding to 286.1 eV. Shirley background was subtracted, and multipeak fits were performed by using CasaXPS software (www.casaxps.com). All spectra were fitted by using a pseudo-Voigt lineshape GL: 70% Lorentzian and 30% Gaussian curves. Pb 4f was fitted by two doublets (Pb^2+^ and Pb^0^) with a fixed spin-orbit split of 4.88 eV and an FWHM of 1.1 eV. Br 3d was fitted with a single doublet with 1.05 eV spin-orbit separation. N 1s was fitted with a single GL curve with an FWHM of 1.45 eV. O 1s was fitted with a single GL with an FWHM of 2.0 eV.

### 2.6 High-Resolution X-ray Diffraction (HRXRD)

Measurements were performed with CuKα_1_ radiation (wavelength λ = 1.54056 Å) using a Smartlab diffractometer (Rigaku) equipped with a Cu rotating anode. High angular resolution was achieved by combining a four-bounce Ge (220) monochromator and a two-bounce Ge 220 analyzer crystal placed in front of the detector. With this setup, specular 2Theta/Theta scans were collected.

## 3 Results and Discussion

### 3.1 Optical Spectroscopy

SPS has been used since the ‘70s to characterize semiconductor surfaces and bulk properties, such as surface potential, minority charge carrier lifetimes, and diffusion lengths. Later, the method has been successfully applied also to the study of interfaces and defect states in semiconductors, due to its ability in the detection of band gap and sub-band gap states. The method is exhaustively explained in several reviews;^26−29^ here, we briefly summarize it. The SPV signal is defined as the illumination-induced variation of the semiconductor surface potential:
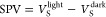
1where *V*_S_^light^ and *V*_S_^dark^ are the surface potentials under illumination and under darkness, respectively. In the spectral region near the fundamental absorption edge and when reflectivity variation is less pronounced than transmittivity (as shown by Brittman et al. for OHPs^30^), the SPV signal is related to the optical absorption coefficient (α)^31^ by
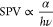
2

Thus, this technique is a remarkable tool to gather the whole absorbance spectrum of semiconducting crystals in a single measurement and without the need of thinning the sample, or relying on two separate measurements such as transmittance below the band gap and reflectance above the band gap.^32,33^ The high surface sensitivity of SPS allows for the detection of defects and sample modifications especially in the near surface region.

**Figure 1:**
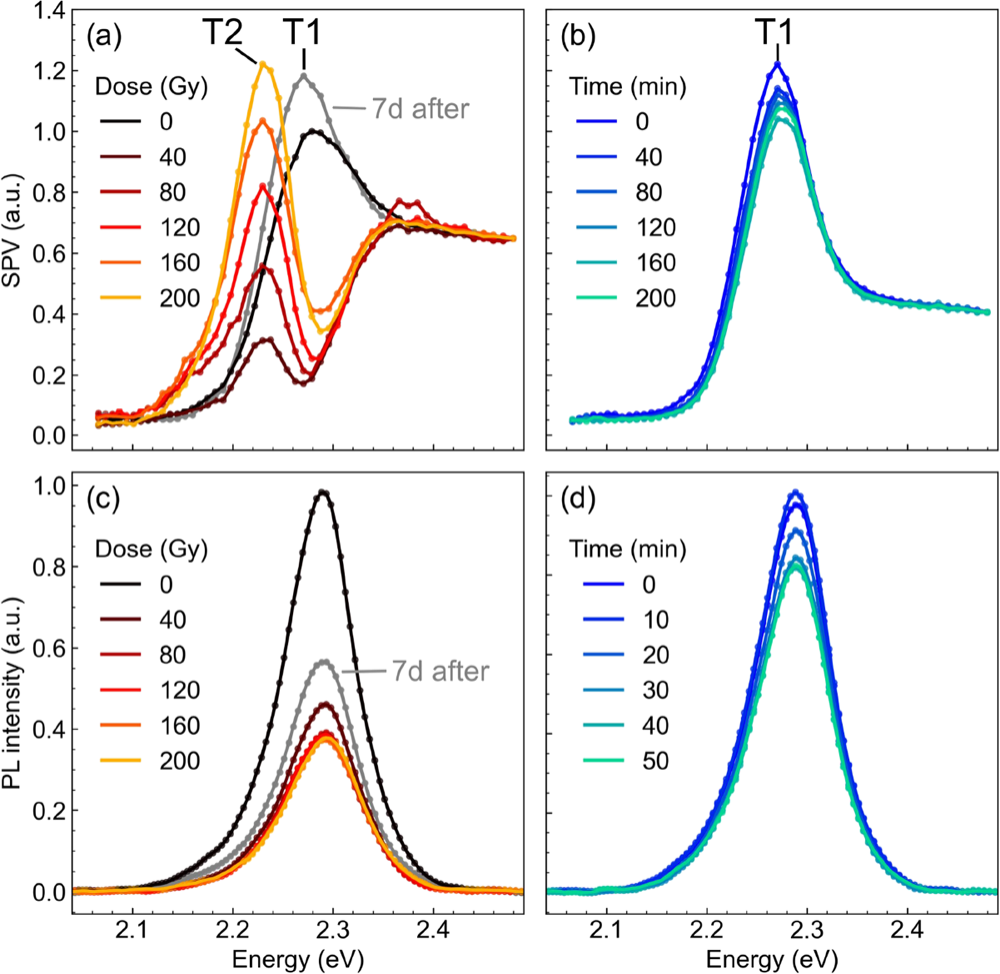
(a,b) Surface photovoltage and (c,d) photoluminescence spectra of an MAPbBr_3_ single crystal as a function of X-ray dose for the (a,c) irradiated sample and as a function of time for the (b,d) control sample. The gray curves in (a,c) show the SPS and PL spectra after 1 week of storage.

To evaluate the effects of X-ray irradiation on the optical absorption properties of an MAPbBr_3_ single crystal, we collected SPS spectra on the as-grown sample and after each step of irradiation with a fixed X-ray dose of 40 Gy air kerma (in the following simply expressed as “Gy).” Irradiation was performed in air with a tungsten (W) target X-ray tube with an accelerating voltage of 150 kV and a tube current of 500 μA. An example of the evolution of the SPS spectrum with the cumulated dose is presented in Figure 1a. The spectrum of the pristine sample (black curve) shows the typical Wannier-Mott excitonic peak (here referred to as T1) generally observed in the absorption spectrum of this material.^34,35^ T1 disappears as soon as the first X-ray dose is delivered, revealing the band-to-band absorption edge and a new peak at lower energies (T2), which increases proportionally to the absorbed dose. This effect cannot be ascribed to sample aging, as demonstrated by the consistency in time of the SPS spectra of a control sample (Figure 1b), which was not exposed to X-ray radiation. The time intervals between successive acquisitions for the control sample were chosen to be the same as for the irradiated sample. This effect is reversible in a timescale of 1 week of storage in dark conditions inside a desiccator with less than 20% humidity (gray curve in Figure 1a). Figure 1c shows the evolution with X-ray dose of the PL spectrum. The PL lineshape is not affected by X-ray exposure, indicating that T2 states are not emissive. The PL intensity instead is significantly affected, with a drop by more than 60%, to be compared with a less than 20% drop in the control sample (Figure 1d). The decrease in PL intensity is consistent with the quenching of the excitonic peak T1. Indeed, in this material, PL at room temperature arises mainly from the recombination of the Wannier-Mott exciton ground state.^34,36−38^ In contrast with SPS, the PL intensity after 1 week (gray curve in Figure 1c) only shows a partial recovery of about 20%, indicating that the photophysical properties of the material are not fully recovered. This might be due to the formation of new chemical species at the surface creating nonradiative deep defect levels. In order to test this hypothesis, we performed SPS measurements in the below-gap spectral range by using a 590 nm long-pass filter to mask any contribution arising from photons with energy higher than the energy gap of the material.^39^ The results are reported in Figure S1. Within the sensitivity of our setup, we did not detect any deep states in the material even after 200 Gy irradiation. We note however that the sensitivity of SPS to deep states is severely limited by the low absorption coefficient of the material in the below-gap spectral region. The study of radiation-induced deep levels will be an object of future work, based on techniques more sensitive to deep levels.

**Figure 2:**
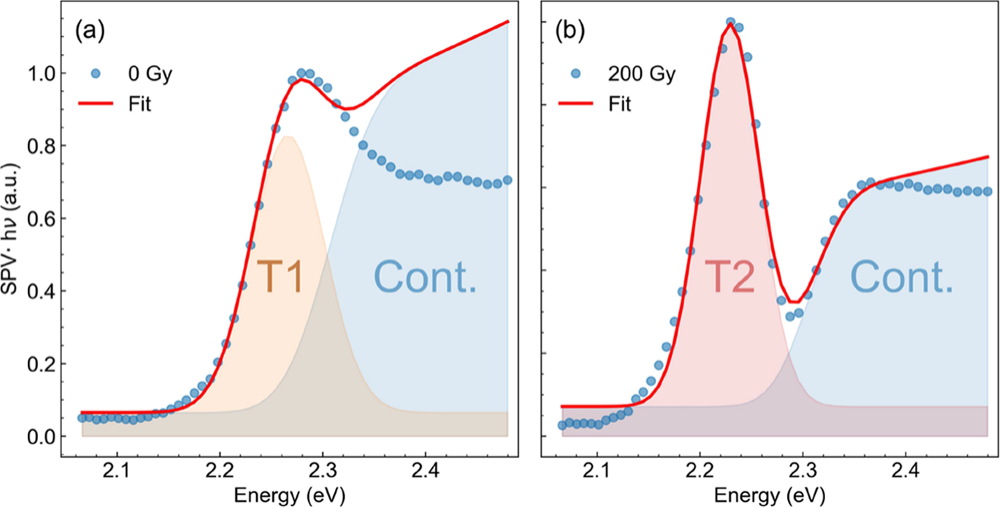
Fitting of SPS spectra by means of Elliott’s formula at (a) 0 Gy and (b) 200 Gy. The blue circles represent the experimental data; the red curve shows the fit result. The blue shaded areas represent the band-to-band (continuum) fit component. The orange and red areas represent the T1 and T2 excitonic fit components, respectively.

According to Sestu et al.^40^ and given the above mentioned direct proportionality between the SPV signal and the absorption coefficient (eq 2), we analyzed our SPS spectra by means of Elliott’s formula.^41^ This formula describes the absorption spectrum of a direct bandgap semiconductor as the sum of two terms: excitonic absorption (characterized by the binding energy *E*_b_) and band-to-band absorption (characterized by the energy gap *E*_g_). Both contributions are phenomenologically convoluted with a gaussian function *g*(*E*) of width Γ, yielding the following equation:^40,42,43^
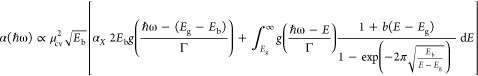
3where *μ*_cv_^2^ is the transition dipole moment and *b*(*E* – *E*_g_) = 10(*m*^2^*E*/*ℏ*^4^)*c*_np_, with *m* being the electronic mass, *ℏ* being the reduced Planck’s constant, and *c*_np_ accounting for nonparabolic conduction and valence bands: *E*(*k*) = *ℏ*^2^*k*^2^/2 *m* – *c*_np_*k*^4^, with *k* being the electronic wavevector. We set *b* = 0.91 eV^–1^ based on typical parameters for MAPbBr_3_.^40^ We introduced the *a_x_* parameter as a scaling factor for the excitonic component with respect to the continuum one, accounting for the scaling of the T2 peak with X-ray dose. We fixed *a*_x_ = 1 for the spectrum at 0 Gy, leading to the standard Elliott formula in the case of a pristine sample. We note that in eq 3, we reported only the ground-state (1s) contribution of the excitonic absorption, i.e., the only one observable at room temperature. In general, the difficulty of fitting experimental absorption spectra by eq 3 arises from the strong convolution of the exciton and continuum part of the spectrum. However, in our spectra after irradiation, the complete quenching of the T1 peak allows us to clearly resolve the continuum absorption. We took advantage of this phenomenon and first used eq 3 to fit the absorption spectrum after irradiation in the energy range of the band-to-band absorption in order to extract the value of the energy gap. We obtained a value of *E*_g_ = 2.30 ± 0.01 eV, in agreement with values reported by other groups.^44,45^ We kept the energy gap fixed to this value in the following fit procedure to reduce as much as possible the number of free parameters. Next, we fitted the spectra in the range 2.1–2.33 eV by letting *E*_b_ and Γ as free parameters. We excluded from the fit the high energy end of the spectrum (above 2.33 eV), where defect-induced surface recombination quenches the SPV signal and eq 2 does not hold.^46^Figure 2 shows the resulting fit (red curve) for the 0 and 200 Gy spectra, as well as the excitonic (orange and red areas) and continuum (light blue areas) components. The fit yielded an exciton binding energy of 39 ± 2 meV at 0 Gy for the T1 peak and of 76 ± 7 meV at 200 Gy for the T2 peak. It can be noted that both these values are comparable with the wide range of values reported in the literature so far.^16,47^

### 3.2 Structural and Compositional Characterizations

To understand whether the X-ray irradiation effects observed in SPS spectra can be ascribed to structural modifications in the sample, we carried out HRXRD analysis. The XRD profiles on the same crystal before and after 200 Gy irradiation (Figure S2) do not show any relevant difference in peak intensity or shape. This clearly evidences that X-ray irradiation does not induce structural modification in the crystal.

**Figure 3:**
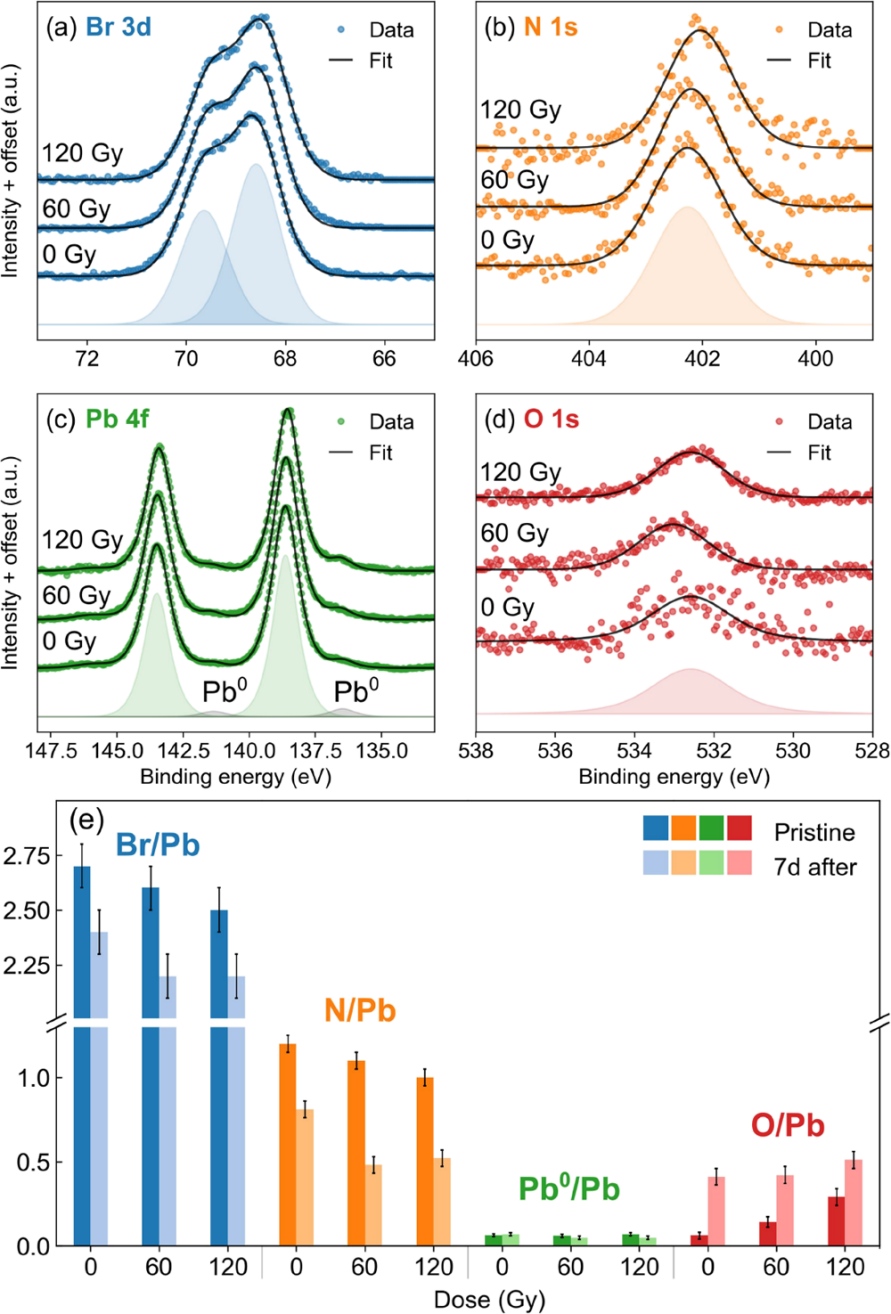
Results of the XPS analysis on the three MAPbBr_3_ single crystals exposed to 0 Gy (control sample), 60 Gy, and 120 Gy X-ray radiation in air. (a–d) XPS spectra of Pb 4f, Br 3d, N 1s, and O 1s. Each graph shows the experimental data as dots and the fitting curve as black solid lines. The components of the fitting curves for the 0 Gy sample are displayed as shaded areas. (e) Elemental ratios with respect to the total amount of Pb calculated from the XPS spectra as a function of absorbed dose, both immediately after irradiation (opaque bars) and after 1 week of storage (semitransparent bars).

Therefore, we moved to a compositional analysis by means of X-ray photoelectron spectroscopy (XPS) to investigate the relative amount and the chemical state of different elements in the surface region, which is expected to be highly sensitive to the chemical interaction in the environment. According to the TPP-2 M formula,^48,49^ the inelastic mean free path of the photoelectrons in the core-level spectra shown in Figure 3 ranges from 1.7 to 2.5 nm, thereby setting the mean probing depth of the XPS measurements around 6–7 nm. This information is complementary to the SPS results, whose probing depth is of the order of few microns. We performed XPS scans on three samples grown from the same precursor solution and exposed to the W target X-ray source in air with increasing dose: 0 Gy (control sample), 60 Gy, and 120 Gy. The measurements were performed immediately after irradiation and repeated after 1 week of storage. In order to minimize X-ray damage due to the XPS source, we took extreme care in limiting as much as possible the collection time of the spectra. To quantify this damage, we performed a calibration experiment by acquiring repeated XPS spectra on a pristine sample. The X-rays used by XPS illuminated homogeneously the entire sample, while the photoelectrons were collected from a 3 mm diameter spot in the middle of the sample. As reported in other studies,^18^ we observed the appearance of a metallic lead (Pb^0^) component in the spectrum and a decrease in the Br/Pb ratio. The results of the calibration experiment are reported and discussed in Figure S3 and Table S1.

Figure 3a–d shows the Pb 4f, Br 3d, N 1s, and O 1s core-level spectra measured for the three samples immediately after irradiation. All spectra were normalized to the same height for better lineshape comparison. In Figure 3e, we report the elemental ratios of the corresponding chemical elements immediately after irradiation (opaque bars) and after 1 week of storage (semitransparent bars). The ratios were calculated relatively to the total amount of Pb using the intensity of the core-level peaks obtained by the fitting analysis of the XPS spectra. The numerical values of the elemental ratios can be found in Table S2. The amount of oxygen, negligible in the control sample, roughly doubles at every dose step, while both Br and N concentrations drop proportionally to the dose. The relative amount of Pb^0^ is 6–7% for all samples, contrary to the increase in metallic lead observed in our calibration experiment and reported in the literature for X-ray exposure inside vacuum chambers.^18^ This suggests that irradiation in air does not produce metallic lead, as opposed to the case of irradiation in a vacuum, probably due to a more complex chemical reaction involving reaction with the environment. Based on these observations, we propose that upon X-ray exposure in environmental conditions, MAPbBr_3_ reacts with oxygen undergoing the chemical reaction
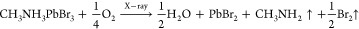


An equivalent degradation process has been proposed by Senocrate et al. for MAPbI_3_.^50^ This reaction occurs also in dark conditions, but high energy radiation is likely to accelerate it through creation of superoxide intermediates O_2_^–^, as reported by other groups in the case of visible light irradiation.^51,52^ Such reaction is consistent with the loss in N, leaving the sample in form of gaseous CH_3_NH_2_, with the loss in Br, forming gaseous Br_2_, and with the formation of water in situ, as indicated by the O 1s peak at 533 eV.^18^ Importantly, we observed T2 in the SPS spectrum even after the XPS calibration experiment, where the sample was irradiated in an ultrahigh vacuum inside the XPS chamber (Figure S4). In the calibration experiment, we detected neither nitrogen loss nor increase in oxygen (Table S1); thus, the only degradation process observed by XPS for both air and vacuum irradiation is the loss of Br. Therefore, we propose bromine vacancies (*V*_Br_) to be responsible for the formation of the T2 species, as discussed in the following. After the XPS scans, all samples were stored under the same conditions described above and measured again after 1 week. The resulting elemental ratios are shown in Figure 3e as semitransparent bars. All samples show a drop in Br and N content, as well as an increase in O concentration, compatible with the degradation process in environmental conditions proposed above, although in dark conditions. The irradiated samples show stronger degradation after 1 week with respect to the control sample, with higher loss in Br and N content. The only process that can be related to a recovery in the material as observed by SPS is the increase in oxygen. Therefore, we propose that environmental oxygen and water fill the bromine vacancies produced by X-ray irradiation, leading to a passivation effect and thus to a recovery of the SPS spectrum.

The creation of *V*_Br_ induced by X-ray irradiation can change the dipole moment in the crystal, as suggested by Anusca et al.,^13^ and thus can affect the dielectric behavior of the material, i.e., the ability of ions in screening electron–hole Coulomb interactions. As perovskites are polar materials, Coulomb interactions among charge carriers are screened by local lattice polarization and transport properties of these crystals should be better discussed in terms of polarons.^16,36^ The coupling of charged carriers with the lattice polarization results in an increase of their effective mass, which can be approximated in the weak coupling regime as^53^
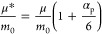
4

With *m_0_* being the free electron mass, μ the bare effective carrier mass (resulting only from band dispersion, neglecting interaction with phonon polarization), μ^*^ the polaronic effective mass, and α_p_ the dimensionless Fröhlich coupling constant.

If we assume that the Bohr model for excitons can be adopted,^36^ the exciton binding energy can be written as
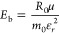
5with *R*_0_ being the Rydberg constant, ε_r_ the effective material dielectric function. From eq 5, we can estimate μ/*m*_0_. By using the dielectric function accounting for ionic screening *ε*_r_ = 0.18 proposed by Sendner et al.^14^ and our experimental result *E*_b_ = 39 meV, we obtained μ/*m*_0_ = 0.09 for the pristine sample, in close agreement with literature values.^54^ The appearance of the T2 transition after irradiation can be attributed to a bound exciton whose effective mass becomes heavier due to the interaction with the lattice, i.e., to polaronic species. If we assume that we can use eq 5 for the estimation of the effective mass μ* of this new species, using a value of *E*_b_ = 76 meV measured by SPS for T2, we obtain μ^*^/*m*_0_ = 0.17. Therefore, the effective mass significantly increases due to exciton–phonon interaction. The relative Frölich coupling constant *a*_p_, as calculated from eq 4, becomes equal to 5.7, much larger than the value of 1.69 reported by Sendner et al. for this material in pristine conditions.^14^ Summarizing, X-ray irradiation creates bromine vacancies in the lattice, which induce a considerable increase in the Frölich coupling constant. While transition T1 is due to free excitons weakly coupled to phonons, transition T2 can be related to excitons strongly coupled with phonons. Lattice deformation and dynamic lattice screening prevent electron–hole radiative recombination for T2, explaining why no T2 contribution is detectable by PL spectroscopy. After 1 week of air exposure, water and oxygen content increases as observed by XPS. We propose that water and oxygen filling helps reducing bromine vacancy concentration, restoring the normal lattice screening and causing the recovery of the T1 excitonic species. This hypothesis is supported by recent results by Shin et al. who observed iodine vacancy filling upon exposure of MAPbI_3_ films to air after intentional vacancy creation.^55^

## 4 Conclusions

We investigated the photophysical properties of MAPbBr_3_ single crystals by surface photovoltage and photoluminescence spectroscopies as a function of X-ray exposure in ambient conditions in the range of interest for medical applications (40–200 Gy). Structural and compositional properties have been investigated to understand the cause of the significant changes in photophysical properties observed after irradiation. The results show that X-ray irradiation induces bromine vacancy formation, which affects the coupling between photogenerated carriers and optical lattice phonons, creating large polarons. The effect is reversible in the timescale of 1 week, thanks to vacancy filling by environmental oxygen and water. This demonstrates that interaction with the environment plays a crucial role in determining the recovery displayed by MAPbBr_3_ single crystals after hard X-ray irradiation.

## Supporting Information Available

Additional experimental details including SPS spectra in the below-gap region before and after X-ray irradiation; high-resolution X-ray diffraction on the MAPbBr_3_ single crystal before and after X-ray irradiation; and XPS calibration experiment and measured elemental ratios before and after X-ray irradiation (PDF)
