# Supplementary material for: X-Ray-Induced Modification of the Photophysical Properties of MAPbBr3 Single Crystals
Source: ACS Appl Mater Interfaces. 2021 Dec 1;13(49):58301–8. doi: 10.1021/acsami.1c16072 (PMC8678983; doi:10.1021/acsami.1c16072)
Supplement: Supplementary file 1 — am1c16072_si_001.pdf [file am1c16072_si_001.pdf]

# Supporting Information

## X-ray induced modification of the photophysical properties of $MAPbBr_3$ single crystals

Giovanni Armaroli[1], Laura Ferlauto[1,2,*], Ferdinand Lédée[1,2], Matilde Lini[1], Andrea Ciavatti[1], Alessandro Kovtun[3], Francesco Borgatti[4], Gabriele Calabrese[5], Silvia Milita[5], Beatrice Fraboni[1], Daniela Cavalcoli[1,*]

1: Department of Physics and Astronomy, University of Bologna, Viale Berti Pichat 6/2, 40127, Bologna, Italy

2: Interdepartmental Center for Industrial Research of the University of Bologna (CIRI-MAM), Viale Risorgimento 2, 40136, Bologna, Italy

3: Institute of Organic Synthesis and Photoreactivity - (CNR-ISOF), Via Gobetti 101, 40129, Bologna, Italy

4: Institute for Nanostructured Material Study (CNR – ISMN), Via Piero Gobetti 101, 40129, Bologna, Italy

5: Institute for Microelectronics and Microsystems (CNR – IMM), Via Piero Gobetti 101, 40129, Bologna, Italy

*: Corresponding author

E-mail addresses: laura.ferlauto@unibo.it (Laura Ferlauto), daniela.cavalcoli@unibo.it (Daniela Cavalcoli).

**SPS spectra in the below-gap region**

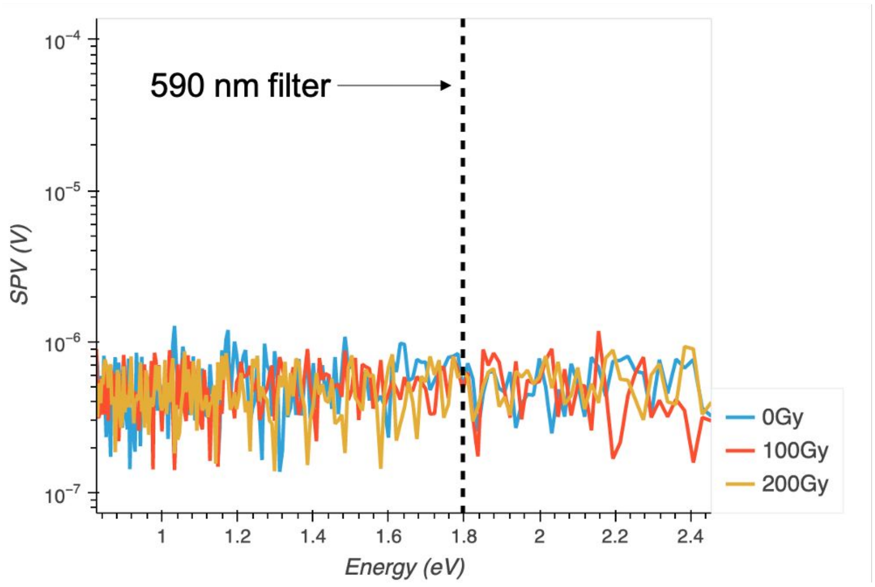


**Figure S1:** SPS spectra in the below-gap region acquired on a pristine sample (light blue curve) and after 100 Gy (red curve), and 200 Gy (yellow curve) irradiation. The vertical dashed line indicates the cutoff wavelength of the 590 nm long-pass filter. Within the sensitivity of the setup, no deep states were detected after irradiation.

**High resolution X-ray diffraction (HR-XRD) on $MAPbBr_3$ single crystal before and after X-ray irradiation**

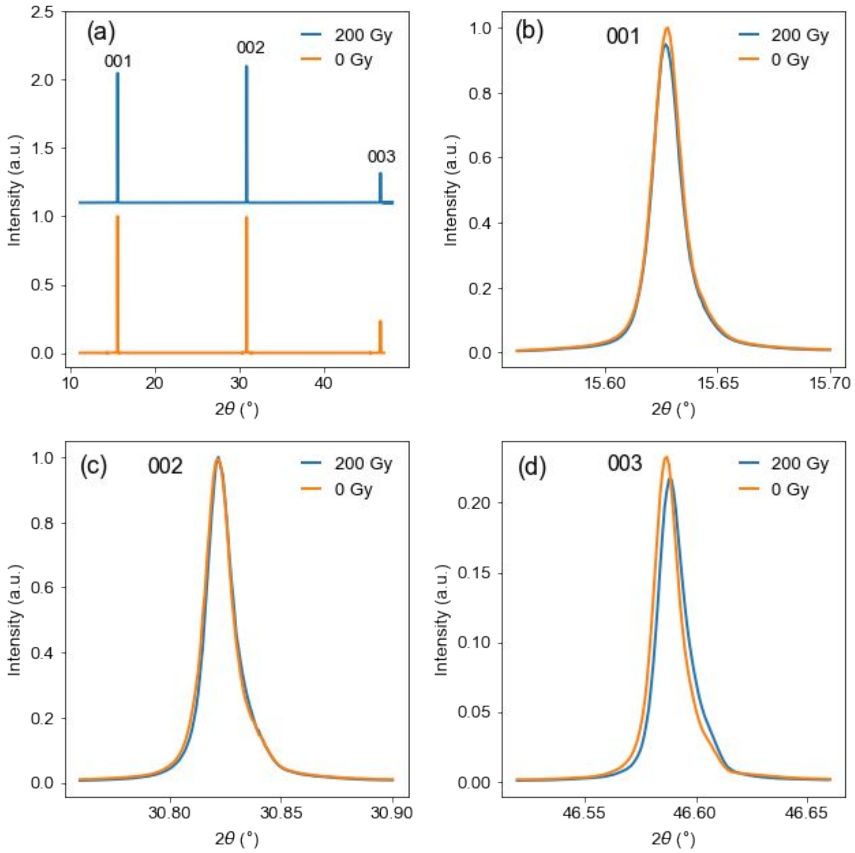


**Figure S2:** (a) High resolution X-ray diffraction spectrum of a $MAPbBr_3$ single crystal before (orange curve) and after (blue curve) 200 Gy irradiation by W target X-ray tube. Detail of 001 (b), 002 (c) and 003 (d) diffraction peaks.

**Description and results of the XPS calibration experiment**

In this experiment we acquired 5 consecutive XPS spectra (duration 1h) on a pristine sample to quantify the effects of the XPS source on the crystals. We observed the appearance of a metallic lead ($Pb^0$) component in the spectrum as well as a decrease in in the Br/Pb ratio, in agreement with previous studies. [1,2] The N/Pb was unchanged during the experiment. These results fit well with the degradation mechanism proposed by Wang et al.[1] under X-rays and in UHV:

$$CH_3NH_3PbBr_3 \xrightarrow{X-ray} Pb^0 + CH_3NH_3Br + Br_2\uparrow \quad \text{in UHV}$$

After the first XPS scan the Pb0/Pb ratio is approximately 7%. Any metallic $Pb^0$ detected below this limit could be due to the XPS source itself rather than to other factors

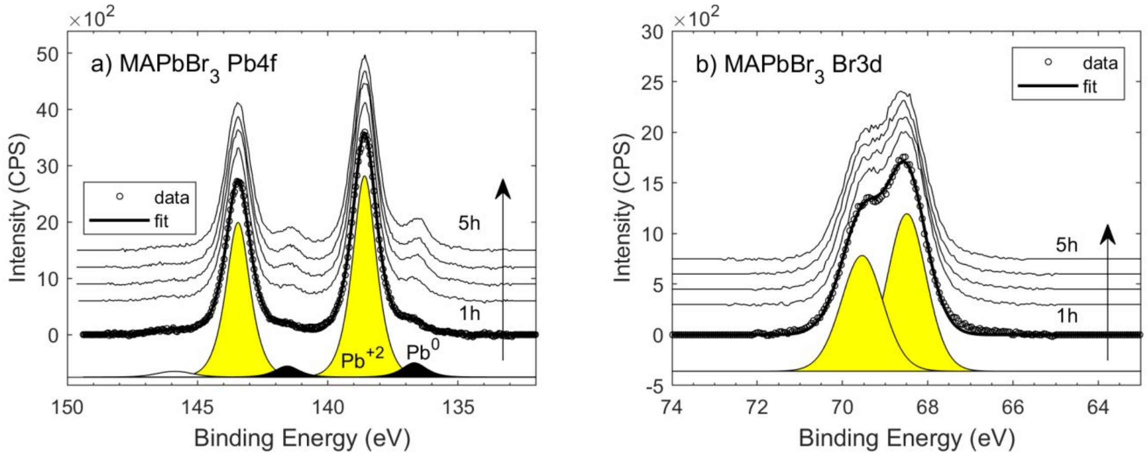


**Figure S3**. Time evolution during XPS calibration experiment to quantify the damage of the XPS beam on the crystals. Pb 4f and $Pb^0$ (a), and Br 3d doublet (b) are reported. The fitting curves are displayed in yellow for $Pb^{+2}$ and Br 3d, and in black for $Pb^0$. An offset was added to the curves for clarity purpose.

| Time | $Pb^{2+} + Pb^0$ | Br | N | $Pb^0$/Pb | O/Pb |
|---|---|---|---|---|---|
| 1h | 1 | 2.8±0.1 | 1.2±0.1 | 0.067±0.009 | 0.04±0.02 |
| 2h | 1 | 2.7±0.1 | 1.2±0.1 | 0.102±0.009 | 0.04±0.02 |
| 3h | 1 | 2.6±0.1 | 1.1±0.1 | 0.127±0.009 | 0.035±0.02 |
| 4h | 1 | 2.6±0.1 | 1.2±0.1 | 0.127±0.009 | 0.03±0.02 |
| 5h | 1 | 2.6±0.1 | 1.2±0.1 | 0.140±0.009 | 0.05±0.02 |

**Table S1.** Elemental ratios in the $MAPbBr_3$ single crystal during XPS calibration experiment. All values are normalized to total Pb = $Pb^0 + Pb^{+2}$ (column 2).

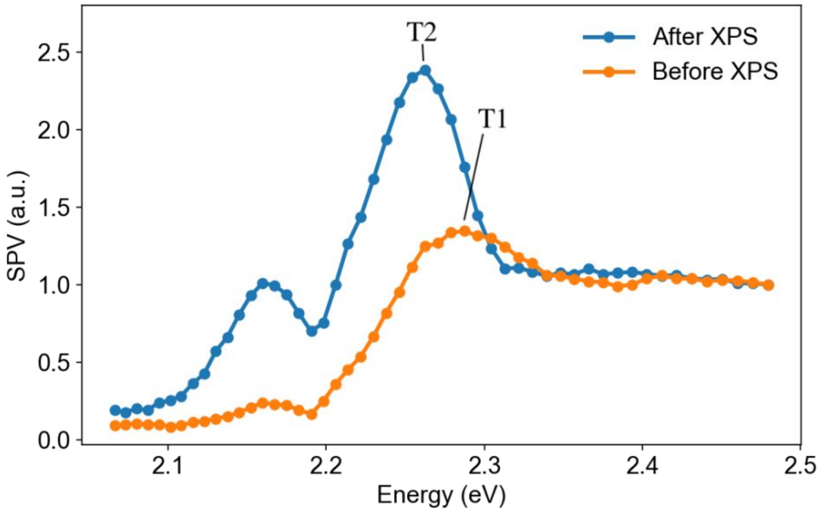


**Figure S4.** SPV spectra of the crystal used for the calibration experiment (orange line) and after (blue line) 4 repeated XPS scans of 1 hour. This shows that quenching of the T1 peak and the appearance of the T2 peak is observed also after irradiation in UHV conditions.

**Elemental ratios as measured by XPS on samples irradiated with W target X-ray source in air**

| Dose (Gy) | Pb | Br | N | $Pb^0$/Pb (%) | O/Pb (%) |
|---|---|---|---|---|---|
| 0 | 1.0 | 2.7 ±0.1 | 1.20 ±0.05 | 6.2 | 6.0 |
| 60 | 1.0 | 2.6 ±0.1 | 1.10 ±0.05 | 5.8 | 14.0 |
| 120 | 1.0 | 2.5 ±0.1 | 1.00 ±0.05 | 6.8 | 29.0 |
| 0 (7 days) | 1.0 | 2.4 ±0.1 | 0.81 ±0.05 | 6.8 | 41.0 |
| 60 (7 days) | 1.0 | 2.2 ±0.1 | 0.48 ±0.05 | 4.7 | 42.0 |
| 120 (7 days) | 1.0 | 2.2 ±0.1 | 0.52 ±0.05 | 4.7 | 51.0 |

**Table S2.** Columns 2-4: relative concentration of Pb, Br and N for samples probed by XPS just after irradiation and after one week. The Pb signal includes both the $Pb^0$ and the $Pb^{2+}$ contributions. Columns 5-6: percentual contribution of $Pb^0$ to the total Pb signal and percentual ratio of oxygen amount with respect to Pb, respectively.

## References


(1) Wang, C.; Ecker, B. R.; Wei, H.; Huang, J.; Gao, Y. Environmental Surface Stability of the MAPbBr3 Single Crystal. *J. Phys. Chem. C* **2018**, *122* (6), 3513–3522. https://doi.org/10.1021/acs.jpcc.7b12740.

(2) Sadoughi, G.; Starr, D. E.; Handick, E.; Stranks, S. D.; Gorgoi, M.; Wilks, R. G.; Bär, M.; Snaith, H. J. Observation and Mediation of the Presence of Metallic Lead in Organic-Inorganic Perovskite Films. *ACS Appl. Mater. Interfaces* **2015**, *7* (24), 13440–13444. https://doi.org/10.1021/acsami.5b02237.
